# Analysis of Polarization Angle on Holographic Recording Based on PQ/PMMA

**DOI:** 10.3390/polym16060821

**Published:** 2024-03-15

**Authors:** Wanxiang He, Defa Liu, Hang Chen, Jundi Wang, Yaping Zhang, Bing Zhang

**Affiliations:** Yunnan Provincial Key Laboratory of Modern Information Optics (LMIO), Kunming University of Science and Technology, Kunming 650500, China; wanxianghe@stu.kust.edu.cn (W.H.); liudefa@stu.kust.edu.cn (D.L.); hangchen@stu.kust.edu.cn (H.C.); jundiwang@stu.kust.edu.cn (J.W.)

**Keywords:** holographic recording, polarization angle, PQ/PMMA photopolymer

## Abstract

The polarization state of light waves significantly affects the quality of holographic recordings. This paper quantitatively analyzes the impact of different polarization states of signal and reference beams on the quality of holographic recordings in PQ/PMMA photopolymer systems during the holography process. By deriving the light field distribution of the interference between two light waves of different polarization states and introducing the interference fringe contrast and the modulation of the refractive index of the photopolymer, we established the relationship between the diffraction efficiency of PQ/PMMA photopolymer holographic gratings and the angle between polarization directions. Based on this relationship, simulations and experiments were conducted. The experimental results demonstrated that as the angle between the polarization directions increased, the diffraction efficiency of the material decreased, with the efficiency dropping to 24.69% of its original value when the angle increased from 0° to 50°. When the angle increased to 60°, the influence of polarization characteristics became gradually significant, and at 90°, it was entirely dominated by polarization characteristics. The photoinduced birefringence properties of the PQ/PMMA prepared in the measurement experiment were studied, and the polarization characteristics of the reconstructed light under polarization direction angles of 0°, 60°, and 90° were investigated. The results indicated that at a polarization direction angle of 60 degrees, the material exhibited a significant response to the polarization information of the signal light. Finally, holographic recordings of objects at different polarization direction angles were conducted, and the reconstructed images were used to visually reflect the impact of the polarization direction angle on the quality of holographic recordings.

## 1. Introduction

Holography is a technique that records the amplitude and phase information of light waves using the principle of interference, first proposed by Gabor [1] in 1948. Early holography employed coaxial optical paths, but due to the low coherence of light sources and the difficulty in separating the conjugate image from the original, the quality of holograms was poor. In 1962 [2], Leith and Upatnieks invented off-axis holography using lasers as the light source, overcoming these limitations and ushering holography into a rapid development phase. The performance of recording media directly affects the results of holographic recording. With ongoing research into holographic recording media, various materials have emerged, including silver halide emulsions, dichromated gelatin, photorefractive crystals, photoresists, and photopolymers [3,4,5,6]. Among these, photopolymers have attracted considerable attention from researchers due to their low production costs, high diffraction efficiency, high photosensitivity, and wide dynamic range [7,8,9,10,11].

Among the various photopolymers studied, phenanthrenequinone-doped polymethyl methacrylate (PQ/PMMA) stands out as a bulk holographic recording material with unique advantages [12]. The preparation process of PQ/PMMA photopolymers is straightforward, involving the separation of the thermal polymerization process from the photopolymerization process, resulting in PQ/PMMA with low photopolymerization-induced shrinkage [13]. Additionally, by varying the mold used in the thermal polymerization process, PQ/PMMA can be produced in various thicknesses, ranging from micrometers to millimeters. PQ/PMMA was initially proposed by Veniaminov in 1991 [14]. Subsequently, in 1996, Veniaminov prepared PQ/PMMA with thicknesses ranging from 100 to 200 μm [7]. In 2004, Hsiao conducted research on the photoresponse mechanisms of PQ/PMMA, identifying the formation of the photoproduct PQ-MMA [15]. Following this, researchers have successively proposed several diffusion models to describe the reaction process of PQ/PMMA [16,17]. As research on PQ/PMMA photopolymers has advanced, it has been discovered that introducing comonomers and doping with nanoparticles can significantly enhance the material’s holographic performance. For example, incorporating the comonomer tetrahydrofuryl methacrylate (THFMA) can improve the solubility of PQ, facilitating further photoreactions and ultimately enhancing the material’s polarization holographic performance [18]. Doping photopolymer materials with TiO_2_ nanoparticles can increase the material’s diffraction efficiency, sensitivity, and reduce photoinduced shrinkage [19]. Moreover, doping PQ/PMMA with graphene oxide nanoparticles has been found to significantly improve the material’s polarization diffraction efficiency [20]. In traditional holographic recording using PQ/PMMA, when the signal light and reference light (laser light being linearly polarized) with a polarization direction angle of 0° interfere, a periodic light field distribution of bright and dark regions is formed on the material’s surface. In the bright areas, the photosensitizer PQ absorbs photon energy, initiating photopolymerization, while the dark areas either do not react or react weakly. The photopolymerization products create a difference in the refractive index between the bright and dark areas, forming a holographic grating [15]. PQ/PMMA exhibits certain polarization-sensitive characteristics, enabling it to respond to the polarization state of the laser [21,22]. The polarization state of the signal and reference lights directly influences the formation of the PQ/PMMA holographic grating. Therefore, quantitatively analyzing the changes in holographic recording quality caused by different laser polarization states during the holographic recording process is meaningful.

Polarization holography, which studies holography under different polarization states, utilizes two laser beams with a polarization angle of 90° (linearly polarized light) to form a periodic distribution of polarization states on the material surface. This technique records the amplitude, phase, and polarization information of the signal light through polarization-sensitive recording media [21]. Polarization holography was first proposed by Lohmann A. W. [23] in 1965. Kuroda et al. [24] introduced a tensor-based theory of polarization holography in 2011, and later, the research group led by Tan [25,26,27] derived and verified the faithful reconstruction and zero reconstruction phenomena based on tensor theory. However, the reconstruction quality of polarization holography is limited by the material’s polarization sensitivity, and typically requires the polarization direction angle to be 90° (linearly polarized light). Considering the traditional holographic performance of PQ/PMMA far exceeds its polarization holography performance [20], studying the impact of polarization states on its holographic recording quality solely at a 90° angle between the signal and reference light polarization directions is insufficient. Zang et al. [28] conducted a detailed investigation into holographic recording and its reconstruction under the conditions where the polarization directions of the signal and reference beams were set at 0° and 90°, respectively. Utilizing a PQ/PMMA photopolymer as the recording medium, a novel polarization multiplexing technique was proposed. However, the holographic recording and reconstruction capabilities of PQ/PMMA photopolymers when the polarization direction angle lies between 0° and 90° remain to be thoroughly explored.

This paper aims to quantitatively analyze the variations in holographic recording of PQ/PMMA photopolymers when the angle between the polarization directions is between 0° and 90°. The paper is organized into several sections: initially, a theoretical analysis is presented, deriving the optical field distribution for the interference of two light waves with different polarization states, providing a theoretical foundation for the study. Subsequently, based on the derived results and incorporating the concepts of interference fringe contrast and the modulation of the photopolymer’s refractive index, the relationship between the diffraction efficiency of PQ/PMMA photopolymer holographic gratings and the angle of polarization direction is established. An experimental verification of this relationship is then conducted. Finally, based on the results of the quantitative analysis, polarization direction angles of 0°, 60°, and 90° are selected for further investigation into the polarization characteristics of holographic recordings. Through this study, we elucidate the relationship between the angle of polarization direction and the diffraction efficiency of holographic gratings, providing theoretical and experimental guidance for optimizing holographic recording materials and techniques.

## 2. Theoretical Analysis

**Figure 1:**
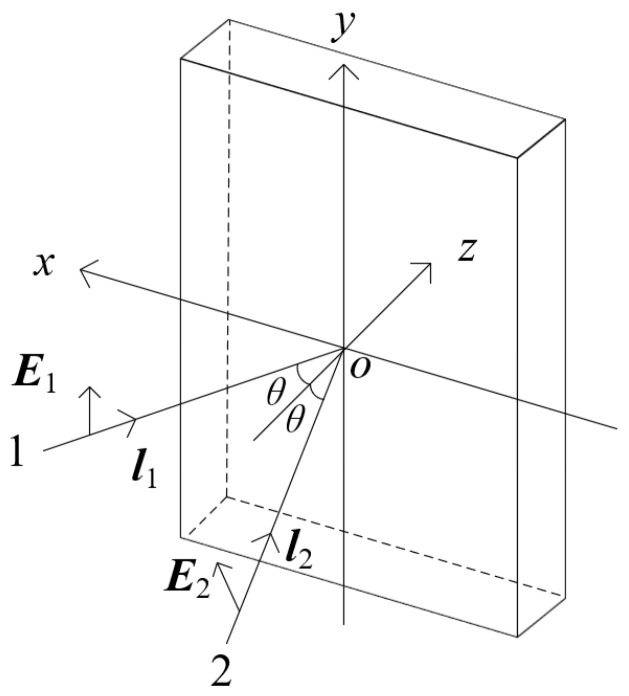
Interference of two plane waves.

For the interference of two plane waves (as shown in Figure 1), light beams 1 and 2 represent two plane waves propagating in the *xoz* plane, with vibration directions $E_{1}$ and $E_{2}$, respectively. These two plane waves interfere on the *xoy* plane, forming an interference grating. We set the vibration direction of light beam 1 to be perpendicular to the *xoz* plane, and the vibration direction of light beam 2 is adjustable, that is,

(1) $$\begin{array}{cccc} & & E_{1}=\left(0,1,0\right) & \end{array}$$

(2) $$\begin{array}{cccc} & & E_{2}=\left(\cos\alpha ,\cos\beta ,\cos\gamma \right) & \end{array}$$

where α, β, and γ are the angles between $E_{2}$ and the *x*, *y*, and *z* axes, respectively. The propagation directions of light beam 1 and light beam 2 are denoted as $l_{1}$ and $l_{2}$, respectively, as shown in Figure 1, θ is the incident angle, and the plane of incidence is on the *xoz* plane.

(3) $$\begin{array}{cccc} & & l_{1}=\left(-\sin\theta ,0,\cos\theta \right) & \end{array}$$

(4) $$\begin{array}{cccc} & & l_{2}=\left(\sin\theta ,0,\cos\theta \right) & \end{array}$$

The complex amplitudes of light beam 1 and light beam 2 on the *xoy* plane are $U_{1}$ and $U_{2}$, respectively. Considering the vibration directions of these two light beams, the following formulas can be derived:

(5) $$\begin{array}{cccc} & & U_{1}=A_{1}n_{y}\cdot e^{i(k_{1}\cdot r_{1}+\varphi_{1})} & \end{array}$$

(6) $$\begin{array}{cccc} & & U_{2}=\left(A_{2}\cos\alpha n_{x}+A_{2}\cos\beta n_{y}+A_{2}\cos\gamma n_{z}\right)\cdot e^{i(k_{2}\cdot r_{2}+\varphi_{2})} & \end{array}$$

where $A_{1}$ and $A_{2}$ are the scalar amplitudes of the two light beams, $n_{x}$, $n_{y}$, and $n_{z}$ are the unit vectors of the x, y, and z axes, respectively, $\varphi_{1}$ and $\varphi_{2}$ are the initial phases of the light waves, and $k_{1}$ and $k_{2}$ are the wave vectors of the light waves. The combined light field distribution on the xoy plane is $U=U_{1}+U_{2}$; thus, the combined light intensity I is expressed as:

(7) $$\begin{array}{cccc} & & I=U^{*}\cdot U=A_{1}^{2}+A_{2}^{2}+2A_{1}A_{2}\cos\beta \cdot \cos\left(k_{1}\cdot r_{1}+\varphi_{1}-k_{2}\cdot r_{2}-\varphi_{2}\right) & \end{array}$$

where $U^{*}$ is the complex conjugate of U.

**Figure 2:**
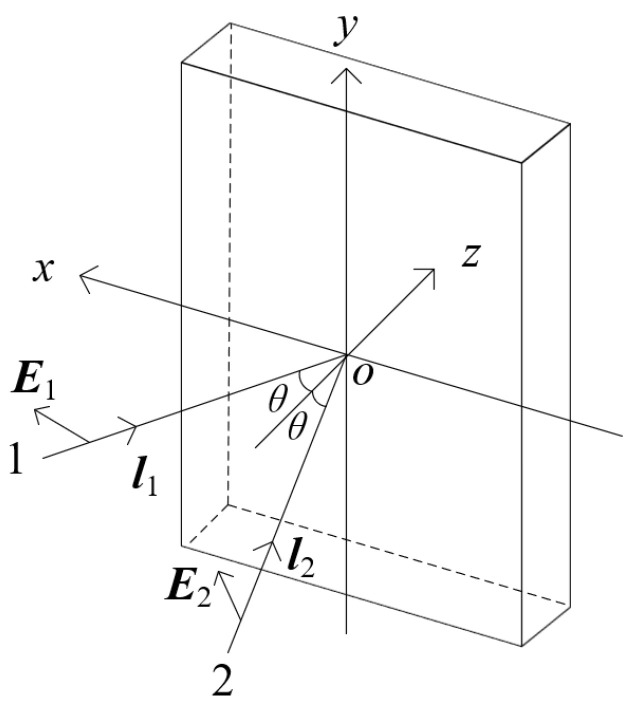
Interference of two plane waves after changing the vibration direction of light beam 1.

We discuss another scenario where the vibration direction of light beam 1 is within the xoz plane (as shown in Figure 2). Thus, the vibration directions and the light field distribution of the two light beams are as follows:

(8) $$\begin{array}{cccc} & & E_{1}=\left(\cos\theta ,0,\sin\theta \right) & \end{array}$$

(9) $$\begin{array}{cccc} & & E_{2}=\left(\cos\alpha ,\cos\beta ,\cos\gamma \right) & \end{array}$$

(10) $$\begin{array}{cccc} & & U_{1}=\left(A_{1}\cos\theta n_{x}+A_{1}\sin\theta n_{z}\right)\cdot e^{i(k_{1}\cdot r_{1}+\varphi_{1})} & \end{array}$$

(11) $$\begin{array}{cccc} & & U_{2}=\left(A_{2}\cos\alpha n_{x}+A_{2}\cos\beta n_{y}+A_{2}\cos\gamma n_{z}\right)\cdot e^{i(k_{2}\cdot r_{2}+\varphi_{2})} & \end{array}$$

In this case, the combined light field distribution on the xoy plane is $U=U_{1}+U_{2}$, and the combined light intensity I is expressed as:

(12) $$\begin{array}{cccc} & & \begin{array}{cc} I & =U^{*}\cdot U\\ & =A_{1}^{2}\cos^{2}\theta +A_{1}^{2}\sin^{2}\theta +A_{2}^{2}\left(\cos^{2}\alpha +\cos^{2}\beta +\cos^{2}\gamma \right)+2A_{1}\cos\theta \cdot A_{2}\cos\alpha \cdot \;cos\\ & \;\left(k_{1}\cdot r_{1}+\varphi_{1}-k_{2}\cdot r_{2}-\varphi_{2}\right)+2A_{1}\sin\theta \cdot A_{2}\cos\gamma \cdot \cos\left(k_{1}\cdot r_{1}+\varphi_{1}-k_{2}\cdot r_{2}-\varphi_{2}\right)\\ & =A_{1}^{2}+A_{2}^{2}+2A_{1}A_{2}\cos\theta \cdot \cos\alpha \cdot \cos\left(k_{1}\cdot r_{1}+\varphi_{1}-k_{2}\cdot r_{2}-\varphi_{2}\right)+2A_{1}A_{2}\sin\theta \cdot \\ & \;\cos\gamma \cdot \cos(k_{1}\cdot r_{1}+\varphi_{1}-k_{2}\cdot r_{2}-\varphi_{2}) \end{array} & \end{array}$$

## 3. Simulation and Experiment

Based on the theories presented in the last section, we simulated the interference grating of two plane waves with different polarization angles. By comparing the quality of simulated gratings under different conditions, we validated our theory. To further validate this conclusion, we chose a photopolymer as our recording medium. We employed two beams with differing polarization states to record and reconstruct using the photopolymer.

### 3.1. Numerical Simulation

Furthermore, we normalized the grating light intensity to obtain the light intensity distribution curve, which intuitively reflects the quality differences of the formed grating under different polarization angles. Subsequently, we introduced the interference fringe contrast V to further quantitatively evaluate the grating quality under different conditions.

For our simulation, we used the more concise Formulas (1)–(7). The initial parameters for the simulation experiment were set to α=0°, β=90°−α, γ=0°, θ=15°. The corresponding interference grating simulation images were obtained by varying α=0°,30°,60°,90°.

**Figure 3:**
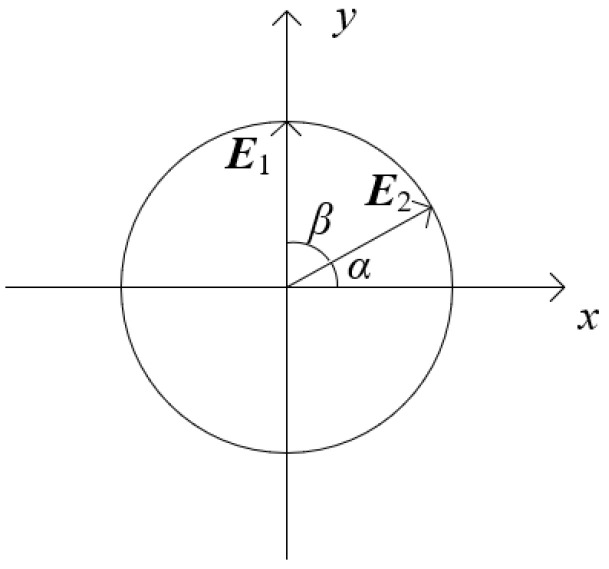
Schematic diagram of the angle between the polarization directions of the light beams.

Since $E_{1}$ is parallel to the *y*-axis, and both $E_{1}$ and $E_{2}$ are perpendicular to the *z*-axis, the angle between the polarization directions of light beam 1 and light beam 2 equals β. The angle of the polarization direction of the beam is shown in Figure 3. β=0° means that the polarization directions of the two light beams are the same, and β=90° means that the polarization directions of the two light beams are orthogonal.

**Figure 4:**
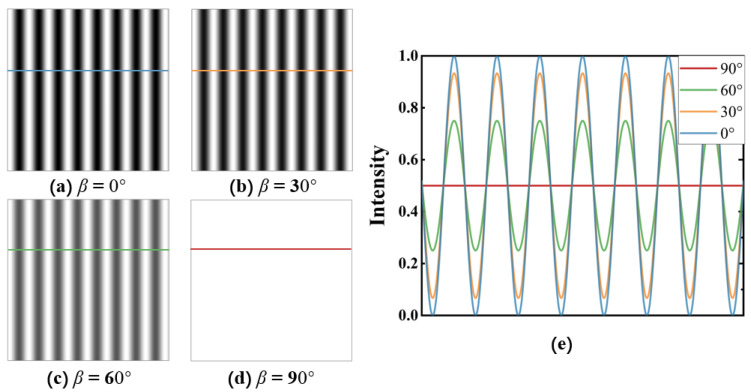
Simulated interference grating images at different polarization angles: (**a**) interference grating when β=0°; (**b**) interference grating when β=30°; (**c**) interference grating when β=60°; (**d**) interference grating when β=90°; (**e**) normalized light intensity distribution curve of the interference grating at different polarization angles.

From Figure 4, we can see that when the polarization directions of the two beams of light are the same (β=0°), the quality of the interference grating is the highest (Figure 4a). As β gradually increases from 0°, the quality of the formed interference grating decreases correspondingly (Figure 4a–c). When the polarization directions of the two beams of light are orthogonal (β=90°), the light intensity is evenly distributed in the area, and no interference grating is formed (Figure 4d). To better reflect the quality of the grating, we took a horizontal line at the same position in each grating, took the maximum light intensity of the bright stripe in the grating at β=0° (Figure 4a) as the standard, and normalized the four gratings. The resulting light intensity distribution curve of the interference grating was obtained and is shown in Figure 4e). The blue curve at 90° in the figure represents the light intensity distribution in the blue horizontal line area in Figure 4a. The meanings of the other curves are similar. As shown in Figure 4e, as the angle β between the polarization directions of the two beams of light gradually increases from 0° to 90°, the gap between the maximum light intensity of the bright stripes and the minimum light intensity of the dark stripes of the corresponding interference grating gradually decreases. This implies that as the angle between the polarization directions of the two beams of light increases, the stripes of the interference grating become more blurred.

To further investigate the variation in the quality of the interference grating, we introduced the contrast of interference fringes (V) as a quantitative measure of the quality of the interference grating. The definition of V was as follows:

(13) $$\begin{array}{cccc} & & V=\frac{I_{\max}-I_{\min}}{I_{\max}+I_{\min}} & \end{array}$$

**Figure 5:**
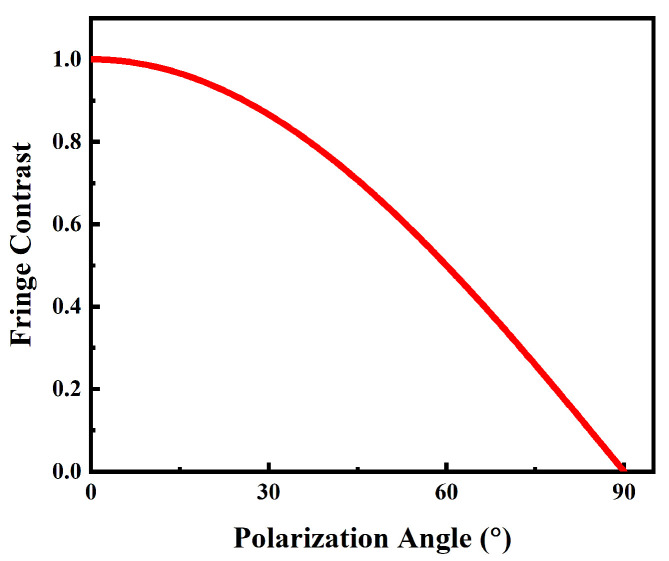
Evolution curve of the contrast of interference fringes (V) with the polarization direction angle β (simulation).

Here, $I_{max}$ and $I_{min}$ represent the maximum and minimum light intensities in the interference fringes, respectively. We investigated the variation in the contrast of interference fringes (V) as β increased from 0° to 90° (as shown in Figure 5).

For the PQ/PMMA photopolymer, the modulation degree of the refractive index can reflect the grating strength. We hypothesized that the refractive index modulation Δn was a function of the fringe contrast V, and by performing a Taylor expansion and disregarding higher-order terms, we obtained:

(14) $$\begin{array}{cccc} & & \Delta n=a\cdot V+b\cdot V^{2}+c\cdot V^{3} & \end{array}$$

In this case, a, b, and c are material-dependent coefficients. According to Kogelnik’s [29] coupled-wave theory, we can determine the relationship between the diffraction efficiency η of the PQ/PMMA photopolymer grating and the refractive index modulation Δn:

(15) $$\begin{array}{cccc} & & \eta =\sin^{2}\left(\frac{\Delta n\pi d}{\lambda \cos\theta_{0}}\right) & \end{array}$$

The material thickness is represented by *d* and the Bragg angle corresponding to the recording light is $\theta_{0}$. Combining Equations (14) and (15), we obtain:

(16) $$\begin{array}{cccc} & & \eta \left(\beta \right)=\sin^{2}\left(\frac{[a\cdot V\left(\beta \right)+b\cdot V^{2}\left(\beta \right)+c\cdot V^{3}\left(\beta \right)]\pi d}{\lambda \cos\theta_{0}}\right) & \end{array}$$

From Equation (16), we can deduce the relationship between the diffraction efficiency η of the PQ/PMMA photopolymer grating and the polarization direction angle β.

### 3.2. Experiment

Building on our simulation results (Figure 4 and Figure 5), we concluded that the quality of the interference grating formed by two beams of light was negatively correlated with the polarization direction angle β(0°≤β≤90°).

The photopolymer material developed in this research consisted of methyl methacrylate (MMA) as the photopolymerizable monomer, phenanthraquinone (PQ) as the photoinitiator, and 2,2-azo-bis-isobutyronitrile (AIBN) as the thermal initiator. Each ingredient was precisely weighed and then combined in a sample vial. The proportion of the mixture was meticulously maintained at MMA:AIBN:PQ = 100:1:1.The sample vial was then placed in an ultrasonic cleaner and subjected to ultrasonic agitation at a temperature of 60 °C for 20 min. Following this, the vial was transferred to a constant temperature water bath and magnetically stirred at 60 °C for approximately 1 h until the mixture became viscous. The viscous solution was subsequently poured into a glass mold with a thickness of 1.5 mm and incubated in a drying oven at 60 °C for 24 h. To terminate the thermal polymerization process, the sample was then refrigerated at 2 °C for a duration sufficient to halt further reaction. Finally, the material was carefully extracted from the glass mold, resulting in the formation of the PQ/PMMA photopolymer.

**Figure 6:**
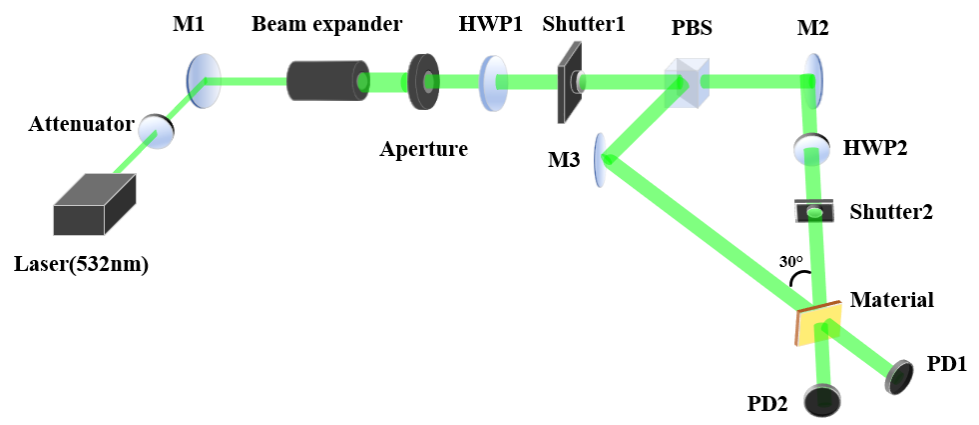
Schematic diagram of the optical path for diffraction efficiency measurement: HWP, half-wave plate; M, plane mirror; PBS, polarizing beam splitter; PD, photodiode power meter sensor.

The optical setup for measuring diffraction efficiency is depicted in Figure 6. We employed a single longitudinal mode semiconductor laser with a wavelength of 532 nm for both holographic recording and reconstruction. The collimated green laser beam was divided into signal and reference beams by a polarizing beam splitter (PBS). The reference beam, reflected towards flat mirror M3 by the PBS, exhibited a vertical polarization (s-polarization), which corresponded to the vibration direction E1 as defined in Equation (1). The signal beam, transmitted through the PBS towards flat mirror M2, was horizontally polarized (p-polarization). The signal beam was then converted to an s-polarization by passing through a half-wave plate (HWP2) oriented such that its fast axis was perpendicular to the horizontal direction (i.e., at an angle of 90°). Both the signal and reference beams had an intensity of 20 mW, and the diameter of the laser beam was 5 mm.

In the process of diffraction efficiency measurement, shutter 1 remained open, while shutter 2 opened for 25 s and then closed for 1 s. This process was repeated 20 times, with PD1 and PD2 recording the corresponding light intensity information. The diffraction efficiency was calculated through the light intensity of the diffracted light and transmitted light during the readout process. The definition of diffraction efficiency η is as follows:

(17) $$\begin{array}{cccc} & & \eta =\frac{I_{1}}{I_{0}+I_{1}} & \end{array}$$

Here, $I_{1}$ represents the diffracted light during the readout process, and $I_{0}$ represents the directly transmitted light.

**Figure 7:**
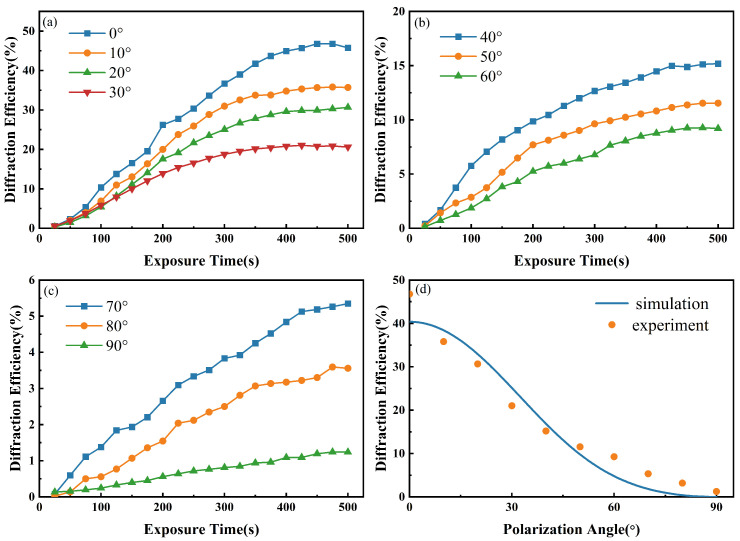
(**a**–**c**) Evolution of the diffraction efficiency over time under different β conditions; (**d**) maximum diffraction efficiency under different β conditions, with the blue curve representing simulated data and the orange data points representing experimental data.

Upon completing a full measurement of the diffraction efficiency, we adjusted the polarization state of the signal light by rotating the half-wave plate (HWP2) by 5°, resulting in a 10° change in the polarization angle of the signal light. This altered polarization state of the signal light was then used for the subsequent measurement of the diffraction efficiency. Throughout all recording phases, the polarization state of the reference light remained unchanged at an s-polarization. As the polarization angle of the signal light varied from 90° (s-polarization) to 0° (p-polarization), the angle β between the polarization directions of the signal and reference beams changed from 0° to 90° to . The diffraction efficiency for different polarization angle β is illustrated in Figure 7.

Figure 7a–c display the evolution of the diffraction efficiency over time under different polarization angle β conditions between signal and reference beams. The orange data points in Figure 7d represent the peak values of each curve in Figure 7a–c, and the blue curve represents the diffraction efficiency curve obtained by simulation based on Equation (16). The impact of the polarization angle β between the signal and reference beams on the diffraction efficiency is significant. As β increases, the diffraction efficiency η decreases from 46.78% to 1.24%.

As discerned from Figure 7d, for the photopolymer PQ/PMMA, the diffraction efficiency η decreases with an increase in the polarization direction angle β. Due to the polarization properties of the PQ/PMMA photopolymer, the influence of the material’s polarization-sensitive characteristics on the diffraction efficiency must be considered when β is not equal to 0°. When β angles are less than 50°, the component of the signal light perpendicular to the polarization direction of the reference light (namely, the p-polarization direction) is minimal, and the holographic grating is primarily determined by the interference of the s-polarization components of the signal and reference lights, forming an intensity grating. Here, intensity gratings refer to gratings formed by signal and reference beams with the same linear polarization state, while polarization gratings are formed by signal and reference beams with differing linear polarization states. As β increases, the intensity in the dark regions of the interference fringes on the PQ/PMMA surface increases, leading to the consumption of the photoinitiator in these areas. The concentration difference between the photoinitiator in the bright and dark areas decreases, reducing the diffusion of the photoinitiator from the dark to the bright areas. This results in a lower-than-expected formation of photo products in the bright areas, which manifests as a faster decrease in the experimental values compared to theoretical expectations. As β gradually increases from 50° to 90°, the component of the signal light in the p-polarization direction increases, and the s-polarization component decreases. This marks the beginning of the manifestation of PQ/PMMA’s polarization-sensitive characteristics, with the holographic grating being influenced by both intensity and polarization gratings. As β approaches 90°, the polarization grating increasingly dominates within the holographic grating. At β = 90°, the interference phenomenon disappears, and the intensity grating becomes ineffective, at which point the holographic grating within the material can be considered to be entirely determined by the polarization grating. The variation in diffraction efficiency with the polarization direction angle, as depicted in Figure 7, illustrates the direct impact of polarization states on the quality of holographic recording in the PQ/PMMA photopolymer. As β increases from 0° to 90°, the inherent polarization characteristics of the material progressively exert a greater influence on the holographic recording, necessitating the measurement of the polarization properties of the PQ/PMMA photopolymer.

Photoinduced birefringence is one of the crucial parameters that illustrates the sensitivity of recording materials to the polarization state of light waves. For PQ/PMMA used as a holographic recording medium, photoinduced birefringence primarily results from the structural reorganization induced by the photochemical reactions of PQ molecules during the holographic recording process [5,22]. To differentiate it from the refractive index modulation (Δn) discussed in this paper, we represent photoinduced birefringence by ΔN. The calculation of the photoinduced birefringence is as follows [21]:

(18) $$\begin{array}{cccc} & & \Delta N=n_{1}-n_{2}=\frac{\lambda }{\pi d}arcsin\sqrt{\frac{I_{T}}{I_{0}sin^{2}2\theta }} & \end{array}$$

In the context of photoinduced birefringence, $n_{1}$ and $n_{2}$ represent the refractive indices of the material parallel and perpendicular to the polarization direction of the pump light, respectively. λ denotes the wavelength of the pump light, d is the thickness of the material, $I_{T}$ is the intensity of the probe light before pump exposure, and $I_{0}$ is the intensity of the probe light after pump exposure. θ is the angle between the pump and probe light.

**Figure 8:**
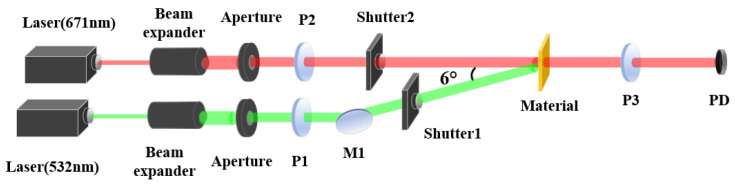
Optical path for measuring photoinduced birefringence: P represents polarizers; M denotes flat plane mirrors; PD stands for the photodetector probe of the power meter.

We conducted a study on the photoinduced birefringence characteristics of PQ/PMMA photopolymers using a single-longitudinal-mode semiconductor laser with a wavelength of 532 nm as the pump light source and another single-longitudinal-mode semiconductor laser with a wavelength of 671 nm as the probe light source. The schematic diagram of the experimental setup is shown in Figure 8. The power of the pump light incident onto the material surface was set to 30 mW with a beam diameter of 5 mm. The probe light was set to have a power of 1 mW incident onto the material surface with a beam diameter of 3 mm. The angle θ between the pump and probe lights was 6°. The polarizers P1, P2, and P3 were oriented at 90° (s-pol), +45°, and −45°, respectively, with the directions of P2 and P3 being perpendicular.

**Figure 9:**
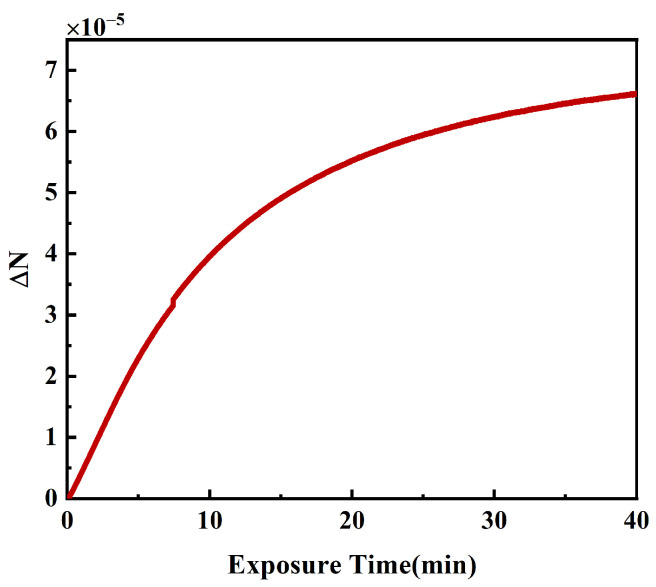
Temporal evolution of photoinduced birefringence.

The results of the photoinduced birefringence in PQ/PMMA with changes in pump light exposure time are depicted in Figure 9. Prior to exposure to the pump light, PQ/PMMA exhibits overall isotropy. Due to the perpendicular orientation of polarizers P2 and P3, the intensity of the probe light on the photodetector (PD) is initially zero. As the pump light exposes the material, the spatial orientation of the photoproducts within PQ/PMMA causes the material to gradually exhibit anisotropy, leading to an increase in the intensity of the probe light detected by PD as the material’s anisotropy strengthens. In the early stages of pump light exposure, photoinduced birefringence increases rapidly due to the high concentration of photosensitive PQ molecules and monomer MMA molecules within the material, facilitating a swift progression of the photoreaction with the accumulation of pump light energy. After a period of pump exposure, most of the PQ molecules in the exposed area are consumed, leading to a decrease in the rate of the photoreaction. Consequently, the rate of increase in photoinduced birefringence becomes slower. The measurement of the photoinduced birefringence parameters of PQ/PMMA demonstrates the ability of our prepared PQ/PMMA photopolymer to respond to the polarization state. It also illustrates that the polarization state can directly affect the outcome of holographic recordings when using PQ/PMMA photopolymers as the recording medium. It is noteworthy that Figure 9 shows that the photoinduced birefringence in PQ/PMMA is not very strong and requires prolonged exposure to the pump light. Therefore, when analyzing the impact of the polarization direction angle on PQ/PMMA holographic recordings, it is necessary to consider both the intensity grating generated by traditional holography interference and the polarization grating from polarization holography theory.

**Figure 10:**
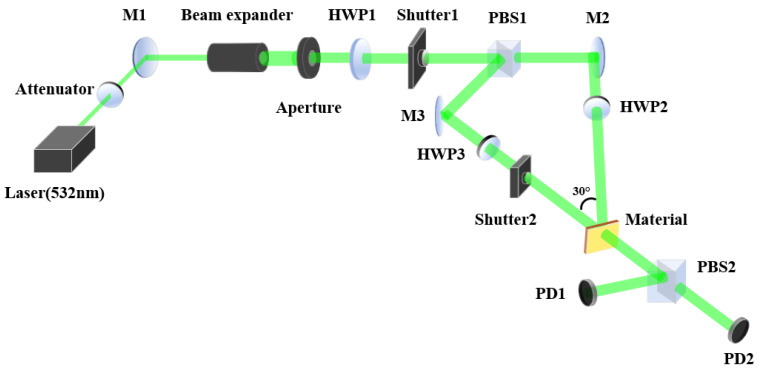
Schematic of the optical path for measuring the reconstructed light’s polarization characteristics.

From Figure 7d, it is observed that at β=60°, the experimental diffraction efficiency of PQ/PMMA is significantly higher than the theoretical value. To further investigate this phenomenon, experiments were designed under three conditions with β set at 0°, 60°, and 90° for holographic recording. Under these recording conditions, the changes in the s-component (s-polarization direction component) and p-component (p-polarization direction component) of the reconstructed light were studied as the polarization direction of the reconstruction reference light was altered. The variation in the s and p components of the reconstructed light reflects the PQ/PMMA photopolymer’s ability to record polarization information. The setup for the experiment is illustrated in Figure 10.

**Figure 11:**
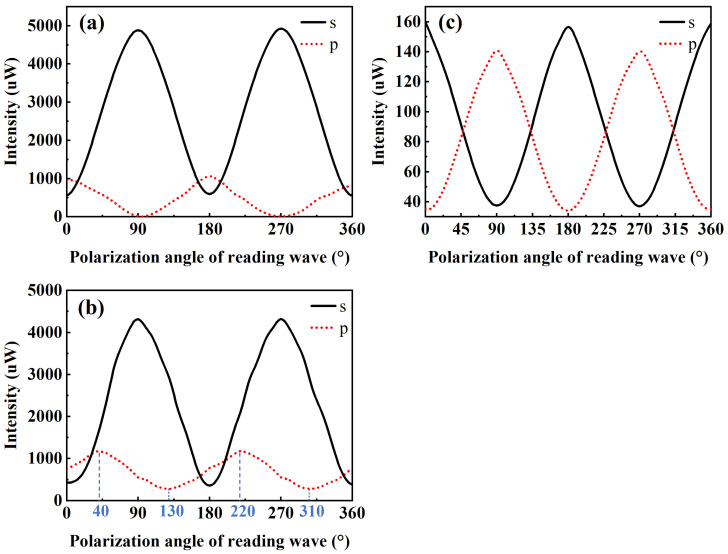
Variation curve of the reconstructed light’s polarization components with the change in the polarization direction of the reconstruction reference light. (**a**) Both signal and reference lights are s-polarized; (**b**) the signal light is polarized at a 30° angle, and the reference light is s-polarized; (**c**) the signal light is s-polarized, and the reference light is p-polarized.

The experimental setup illustrated in Figure 10 is similar to the one shown in Figure 6, utilizing the same 532 nm light source, angle between the signal and reference beams, beam diameter, and laser power incident onto the material surface. However, a key difference is the configuration of the signal and reference lights after passing through PBS1: the light reflected by PBS1 and passing through HWP3 serves as the signal light, while the light transmitted through PBS1 and HWP2 serves as the reference light. During the recording phase, the polarization directions of the signal and reference lights were modified by adjusting the angles of HWP2 and HWP3, respectively. The experiment investigated the polarization characteristics of the reconstructed light from holographic recordings made with signal and reference light polarization direction angles β of 0°, 60°, and 90°. The polarization information for the signal and reference lights during the recording phase were as follows: (1) both signal and reference lights were s-polarized; (2) the signal light was polarized at a 30° angle, and the reference light was s-polarized; (3) the signal light was s-polarized, and the reference light was p-polarized. During the reconstruction process, rotating HWP2 allowed for the generation of reconstruction reference light with any polarization direction. The polarizing beam splitter (PBS2) separated the reconstructed light into s- and p-polarized components. Photodetectors PD1 and PD2 recorded the intensities of the two polarized components of the reconstructed light, facilitating the analysis of how different polarization states of the reconstruction reference light affected the polarization state of the reconstructed light. The results are presented in Figure 11.

Figure 11a represents the variation in the s and p components of the reconstructed light with the polarization direction of the reconstruction reference light, when both the signal light and the reference light used for recording are polarized in the s-pol direction. The s component of the reconstructed light reaches its maximum value, and the p component reaches its minimum value when the reconstruction reference light is polarized at 90° and 270° (i.e., in the s-pol direction), making the polarization direction of the reconstructed light consistent with that of the signal light. Conversely, when the polarization direction of the reconstruction reference light is at 0°, 180°, and 360°, the s component of the reconstructed light reaches its minimum value, and the p component reaches its maximum value. The total intensity of the reconstructed light is very low, which is in accordance with the theoretical derivation results of the polarization holography tensor theory [28]. This indicates that when the angle β between the polarization directions of the signal light and the reference light is 0°, the holographic grating of the PQ/PMMA photopolymer is mainly influenced by the intensity grating formed by traditional holography. The polarization characteristics of the material are reflected in the variation in the p component. Figure 11b depicts the variation in the s and p components of the reconstructed light with the polarization direction of the reconstruction reference light, following the recording with a reference light polarized in the s-pol direction and a signal light polarized at 30°. It is observed that when the angle between the polarization directions of the signal light and the reference light is 60°, the variation pattern of the s component of the reconstructed light remains essentially unchanged with the change in the reconstruction reference light, while the variation pattern of the p component changes. The angle of the reconstruction reference light polarization at which the p component reaches its maximum (or minimum) intensity increases by 40° compared to Figure 11a. This phenomenon is influenced by two factors: first, the component of the signal light in the p-pol direction creates a polarization grating with the reference light, which is recorded by the photoinduced birefringence properties of the PQ/PMMA. Second, the component of the signal light in the s-pol direction interferes with the reference light to produce an intensity grating. Figure 11c illustrates the variation in the s and p components of the reconstructed light with the change in the polarization direction of the reconstruction reference light, following the recording with a signal light polarized in the s-pol direction and a reference light polarized in the p-pol direction. When the angle of the reconstruction reference light is at 0°, 180°, and 360°, the s component of the reconstructed light reaches its maximum value, and the p component reaches its minimum value. At this point, the polarization direction of the reconstructed light is in the s-pol, consistent with the signal light, representing a faithful reconstruction in polarization holography. Conversely, when the angle of the reconstruction reference light is at 90° and 270°, the s component of the reconstructed light reaches its minimum value, and the p component reaches its maximum value, in accordance with the orthogonal reconstruction observed in linear polarization holography [30].

Comparing Figure 11a–c, when the angle β between the polarization directions of the signal and reference lights is 60°, the reconstructed light is primarily influenced by the intensity grating, yet it demonstrates a response to the polarization component of the signal light perpendicular to the reference light’s polarization direction. Additionally, at this angle, the intensity of the reconstructed light is significantly higher than at β=90°, indicating that a brighter and clearer holographic reconstruction image can be obtained. Although at β=60°, the recorded polarization information is greatly affected by the intensity grating, the curve in Figure 7d suggests that as β continues to increase beyond 60°, the diffraction efficiency, which represents the quality of the intensity grating, continues to decrease. Figure 11c shows that at β=90°, polarization information can be well recorded, but the overall intensity of the reconstructed light is very low. Therefore, it may be beneficial to experiment with β angles greater than 60° but less than 90° to achieve a balance where polarization information is adequately recorded while maintaining a diffraction efficiency superior to that observed under conditions where β=90°.

**Figure 12:**
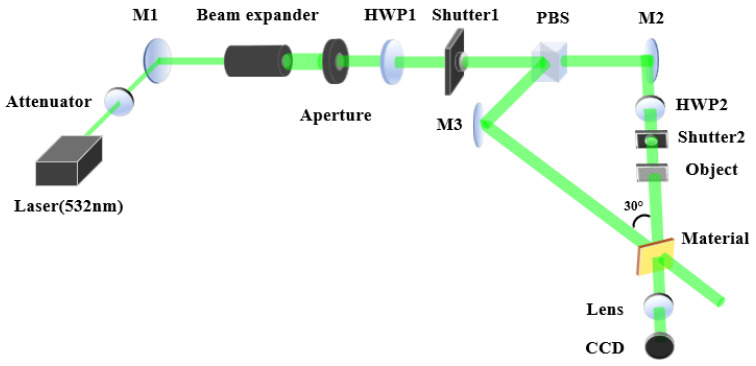
Optical path for holographic recording and reconstruction: HWP, half-wave plate; M, plane mirror; PBS, polarizing beam splitter; Object, transmissive resolution chart.

To more directly observe the impact of the polarization angle β between the signal and reference light on holographic performance, we carried out a holographic recording and reconstruction of a transmissive resolution chart under varying β conditions. The experimental optical path is depicted in Figure 12.

**Figure 13:**
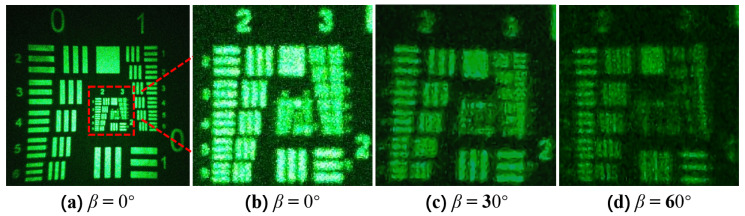
Reconstructed images recorded under different polarization direction angle β conditions: (**a**) the reconstructed image recorded at β=0°; (**b**) the magnified image of the reconstructed image recorded at β=0°, with the magnification area indicated by the red box; (**c**) the magnified image of the reconstructed image recorded at β=30°; (**d**) the magnified image of the reconstructed image recorded at β=60°.

The object for holographic recording is a transmissive resolution chart, recorded under the conditions where the polarization direction angle β is 0°, 30°, and 60°, respectively, and reconstructed with the original reference light. The reproduced image was captured using a CCD (as shown in Figure 13).

Figure 13 shows the reconstructed images recorded under different conditions of polarization direction angle β. Figure 13a is the reconstructed image recorded at β=0°, with a magnified view of the area indicated by the red box shown in Figure 13b. Applying the same operation to the reconstructed images recorded under other conditions results in Figure 13c,d. It can be seen that when β=0°, the numbers “2” and “3” at the top of the reconstructed image are clear, and the stripes at the bottom right are distinct. At β=30°, the numbers “2” and “3” at the top become blurred, the clarity of the stripes at the bottom right diminishes, but they are still discernible. At β=60°, the numbers “2” and “3” at the top become very blurry, the clarity of the stripes at the bottom right continues to degrade, the horizontal stripes remain discernible, but the vertical stripes are indistinguishable. Comparing Figure 13b–d, it can be seen that the quality of the reconstructed image noticeably decreases as β increases, which aligns with the conclusions presented earlier in the paper.

## 4. Conclusions

This paper quantitatively analyzed the changes in holographic recording quality induced by varying the polarization states of the signal and reference beams in PQ/PMMA photopolymers, establishing the relationship between the diffraction efficiency of PQ/PMMA photopolymer holographic gratings and the angle between polarization directions. Experimental results indicated that the holographic diffraction efficiency decreased as the angle between polarization directions increased, with the efficiency dropping from 46.00% to 1.24% as the angle increased from 0° to 90°. The photoinduced birefringence property of the PQ/PMMA polymers we prepared was measured, showing a birefringence of $6.63\times 10^{-5}$ after 40 min of exposure. The polarization characteristics of the reconstructed light under 0°, 60°, and 90° polarization direction angles were investigated. The formation of holographic gratings within the material was influenced both by the intensity distribution of interference fringes and by polarization characteristics. As the angle between polarization directions increased, the influence of the interference fringe intensity distribution on the formation of holographic gratings decreased, while the influence of polarization characteristics increased. When the polarization direction angle was less than 50°, the material was primarily influenced by the intensity distribution of interference fringes. As the angle gradually increased from 50° to 90°, the influence of polarization characteristics on the holographic gratings became more pronounced. At a polarization direction angle of 60°, the experimental holographic diffraction efficiency was significantly higher than theoretical values, and the p-component of the reconstructed light underwent significant changes, proving that the material responded to the polarization information of the signal light. By exploring polarization direction angles between 60° and 90°, there is potential to record the polarization information of the signal light while achieving a diffraction efficiency higher than that of traditional polarization holography (β= 90°).

## Data Availability

The data that support the results within this paper and other findings of the study are available from the corresponding authors upon reasonable request.
